# Elevated progesterone on the trigger day does not impair the outcome of Human Menotrophins Gonadotrophin and Medroxyprogesterone acetate treatment cycles

**DOI:** 10.1038/srep31112

**Published:** 2016-08-08

**Authors:** Xuefeng Lu, Qiuju Chen, Yonglun Fu, Ai Ai, Qifeng Lyu, Yan Ping Kuang

**Affiliations:** 1 Department of Assisted Reproduction, Shanghai Ninth People’s Hospital, Shanghai Jiaotong University School of Medicine, 639 Zhizaoju Rd, Shanghai 200001, China

## Abstract

To demonstrate the incidence and effects of elevated progesterone (P) on the trigger day on the outcome of *in-vitro* fertilization (IVF)/intracytoplasmic sperm injection (ICSI) cycles using Medroxyprogesterone acetate (MPA) co-treated with Human Menotrophins Gonadotrophin (hMG + MPA), we performed a retrospective analysis including 4106 IVF/ICSI cycles. The cycles were grouped according to the P level on the trigger day: <1 ng/mL, between 1–1.5 ng/ml (including 1), between 1.5–2 ng/mL (including 1.5), and ≥2 ng/mL. The primary outcome measure was live birth rate. The prevalence of P level categories was 12.93% (531/4106), 2.92% (120/4106), and 1.92% (79/4106) in women with P between 1–1.5 ng/mL, between 1.5–2 ng/mL, and ≥2 ng/mL, respectively. The mean stimulation duration, total hMG dose, serum follicle stimulating hormone (FSH), estrogen(E2) on the trigger day and the number of oocytes in patients with elevated P were significantly higher than patients with P < 1 ng/mL (P < 0.05). However, there were no significant differences in the oocyte retrieval rates, fertilization rates, implantation rates, clinical pregnancy rates and live birth rates between the groups based on frozen embryo transfer (FET). We concluded that elevated P on the trigger day had no negative effect on the final outcome of the hMG + MPA treatment cycles based on FET.

Subtle increases in serum progesterone (P) levels at the end of the follicular phase in controlled ovarian stimulation (COS) cycles are a frequent phenomenon in *in-vitro* fertilization (IVF)/intracytoplasmic sperm injection (ICSI) treatment cycles despite the use of gonadotropin-releasing hormone (GnRH) agonists and antagonists^1^. Elevated P levels on the day of trigger have been reported with a markedly varied incidence (5% to 30%)^1^,^2^ due to divergences in the definition, population characteristics and/or treatment protocols.

The possible effects of these subtle elevations of P are controversial. Several studies have shown that high serum P levels on the day of trigger could adversely affect the oocyte quality and thus have negative effects on the fertilization rate, embryo quality, and pregnancy rate^3^,^4^. Some studies have found that elevated P levels might impair endometrial receptivity rather than oocyte quality and thus have a negative effect on the pregnancy rate^2^,^5^,^6^. Other studies have indicated that elevated P on the day of trigger did not have any negative effects on the outcome of the IVF/ICSI treatment cycles^7^,^8^,^9^. A recent report showed that elevated P levels on the day of trigger during the initial fresh cycle were negatively associated with live births in the fresh transfer cycles but not in subsequent frozen embryo transfer (FET) cycles^10^.

The mechanism by which the serum P subtly increases at the end of the follicular phase in COS cycles is still controversial. Excessive amounts of P are thought to be produced by prematurely luteinized granulosa cells^11^. However, elevated P cannot be explained by the luteinization of granulosa cells because this process occurs in the presence of GnRH agonists and antagonists and under low serum LH concentrations^7^,^12^. It is more likely that the elevated P levels might be due to an excess number of oocytes, exposure to large amounts of exogenous gonadotropins, or increased LH sensitivity of the granulosa cells^13^.

Recently, we found that Medroxyprogesterone acetate (MPA) was an effective oral alternative for the prevention of premature LH surges in women undergoing COS for IVF^14^. The pregnancy outcomes from FET indicated that the embryos originating from hMG + MPA treatment cycles showed a similar developmental potential to embryos originating from the short protocol. Compared with GnRH antagonists, MPA has the advantages of an oral administration route and providing easy access and more control over the LH levels. In hMG + MPA treatment cycles, the pituitary has not been desensitized by the use of a GnRH agonist or antagonists. The duration of gonadotropin administration and the hMG doses were higher in the MPA cotreatment group than under the short protocol. We observed that elevated P on the trigger day also occurred in the hMG + MPA treatment cycles. When a negative association between P elevation on the day of trigger and the probability of pregnancy exists, it is important to administer the trigger at an earlier time point in the follicular phase prior to P elevation. However, the incidence and effect of P elevation on the day of trigger when using the hMG + MPA protocol are still unknown. This retrospective study was undertaken to answer these questions by analyzing a large population of IVF/ICSI cycles treated using the hMG + MPA protocol (n = 4106). The live birth rate was used as the primary outcome measure.

## Results

### Elevated P incidence

According to the most reports, serum P levels ≥1.0 ng/mL on the day of trigger are defined as a premature P increase^1^. However, elevated P levels defined as ≥1.5 ng/mL or ≥2 ng/mL have been proposed to have a stronger effect on achieving pregnancy than elevated P levels defined as >1 ng/mL^15^,^16^. To evaluate the effect of elevated P on the day of trigger, the cycles were divided into four groups according to the serum P concentrations on the day of trigger: P < 1 ng/mL, P between 1–1.5 ng/mL (including 1), P between 1.5–2 ng/mL (including 1.5), and P ≥ 2 ng/mL. Serum P levels on the day of trigger between 1–1.5 ng/mL were detected in 12.93% of the cycles (531/4106), whereas P values between 1.5–2 ng/mL and ≥2 ng/mL were found in only 2.92% (120/4106) and 1.92% (79/4106) of the cycles, respectively. In total, 17.78% (730/4106) of the cycles showed elevated P (≥1 ng/mL), indicating that subtle P elevation was a frequent phenomenon in hMG + MPA treatment cycles.

### Patient characteristics

A comparison of the patient characteristics indicated that the average age of the patients with P < 1 ng/mL was 31.48 years, which was significantly older than the patients with P values between 1–1.5 ng/mL, 1.5–2 ng/mL and ≥2 ng/mL (31.48 VS 30.56, P < 0.001; 31.48 VS 30.67, P = 0.014; and 31.48 VS 30.18, P = 0.001, respectively) (Table 1). Moreover, patients with P < 1 ng/mL showed significantly lower basal LH levels (3.45 VS 4.08, P < 0.001; 3.45 VS 4.21, P < 0.001; and 3.45 VS 4.54, P = 0.031, respectively) and higher basal FSH levels on stimulation day 1 than patients with P between 1–1.5 ng/mL, between 1.5–2 ng/mL and ≥2 ng/mL (5.79 VS 5.22, P < 0.001; 5.79 VS 4.89, P < 0.001; and 5.79 VS 4.77, P < 0.001, respectively) (Table 1). However, no significant between-group differences existed in terms of basal E2 and P levels (Table 1).

### Ovarian stimulation characteristics and outcomes

**Figure 1:**
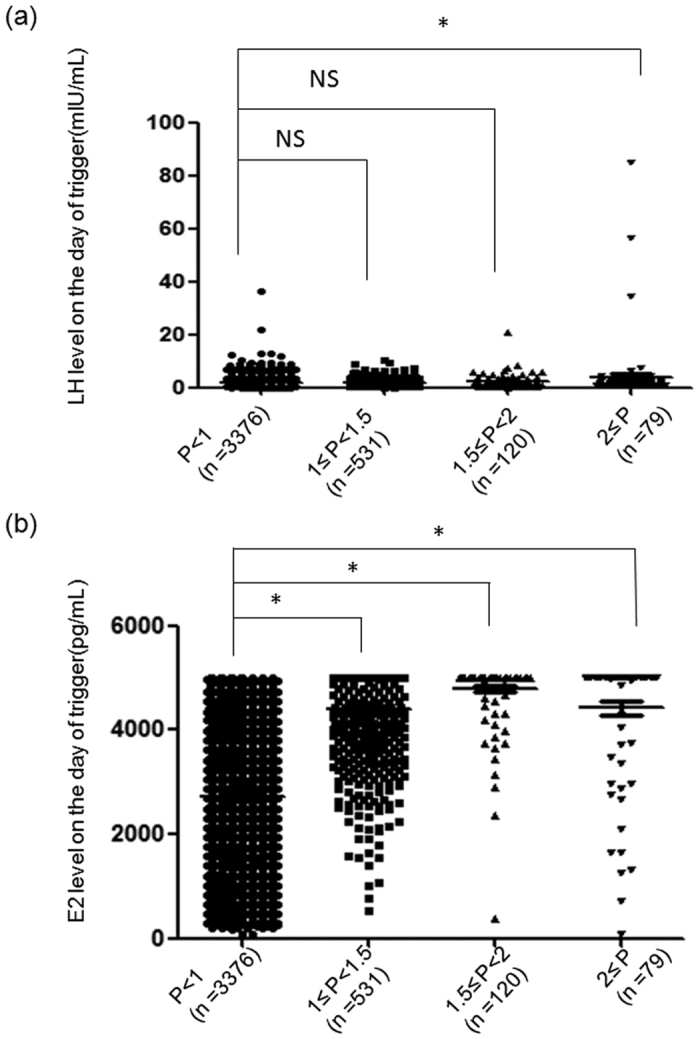
The hormones on day of trigger. (**a**) The LH levels on the day of trigger in the groups with P < 1 ng/mL, P between 1–1.5 ng/mL, P between 1.5–2 ng/mL and P ≥ 2 ng/mL. (**b**) E2 levels on the day of trigger in the groups with P < 1 ng/mL, P between 1–1.5 ng/mL, P between 1.5–2 ng/mL and P ≥ 2 ng/mL. The asterisks indicate P < 0.05, NS: no significant difference.

The ovarian stimulation characteristics of women with P < 1 ng/mL, between 1–1.5 ng/mL, between 1.5–2 ng/mL, and ≥2 ng/mL on the day of trigger are presented in Table 2. The mean stimulation duration and total hMG dose in the groups with P between 1–1.5 ng/mL, between 1.5–2 ng/mL and ≥2 ng/mL were significantly higher than those in the group with P < 1 ng/mL (P < 0.001 for all). The mean FSH and E2 levels on the trigger day in the groups with P between 1–1.5 ng/mL, between 1.5–2 ng/mL and ≥2 ng/mL were significantly higher than those in the group with P < 1 ng/mL (P < 0.001 for all) (Table 2). There were no significant differences in the mean LH level on the trigger day in the groups with P < 1 ng/mL, between 1–1.5 ng/mL, and between 1.5–2 ng/mL. However, we noted that the mean LH level on the trigger day in the group with P≥2 ng/mL was higher than the mean level in the group with P < 1 ng/mL (3.98VS 1.74 P < 0.001) (Table 2 and Fig. 1(a)). However, there was no significant difference in the LH level on the trigger day compared with the basal LH values in the group with P ≥ 2 ng/mL (3.98 VS 4.54. P > 0.05). Moreover, we noted that the LH level was lower than 10 mIU/mL on the trigger day in all patients and thus was not sufficient to induce premature luteinization with the exception of 12 patients, including 7 patients with P < 1 ng/mL, 1 patient with P = 1.1 ng/mL, 1 patient with P = 1.5 ng/mL, and 3 patients with P ≥ 2 ng/mL on the trigger day (Fig. 1(a)). These data suggested that the elevated P levels observed at the end of the follicular phase during hMG + MPA treatment cycles were not due to premature LH surge because the elevation of the P level detected on the trigger day was not accompanied by increased LH.

**Figure 2:**
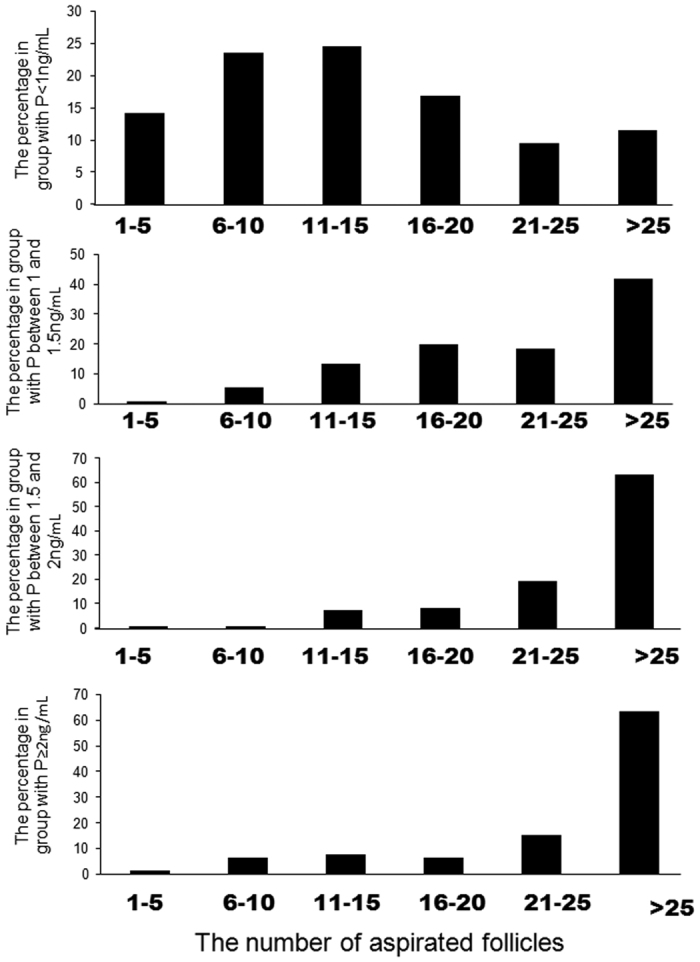
The frequency distribution of number of aspirated follicles in the groups with P < 1 ng/mL, P between 1–1.5 ng/mL, P between 1.5–2 ng/mL and P ≥ 2 ng/mL on the day of trigger.

The numbers of oocytes retrieved were also significantly more in the groups with P between 1–1.5 ng/mL, P between 1.5–2 ng/mL and P ≥ 2 ng/mL than the group with P < 1 ng/mL (19.84 VS 10.38, P < 0.001; 20.39 VS 10.38, P < 0.001; and 17.99 VS 10.38, P < 0.001, respectively) (Table 2). Consistent with these results, E2 levels were significantly higher in the groups with P between 1–1.5 ng/mL, P between 1.5–2 ng/mL and P ≥ 2 ng/mL than those in the group with P < 1 ng/mL (Fig. 1(b)). Further analysis showed that most of the patients with elevated P displayed a high ovarian response to hMG stimulation with more than 15 aspirated follicles (Fig. 2). Only 0.75% (4/531), 0.83% (1/120), and 1.27% (1/79) of the patients had 1–5 follicles aspirated at the end of stimulation in the groups with P between 1–1.5 ng/mL, between 1.5–2 ng/mL and ≥2 ng/mL, respectively (Fig. 2).

**Figure 3:**
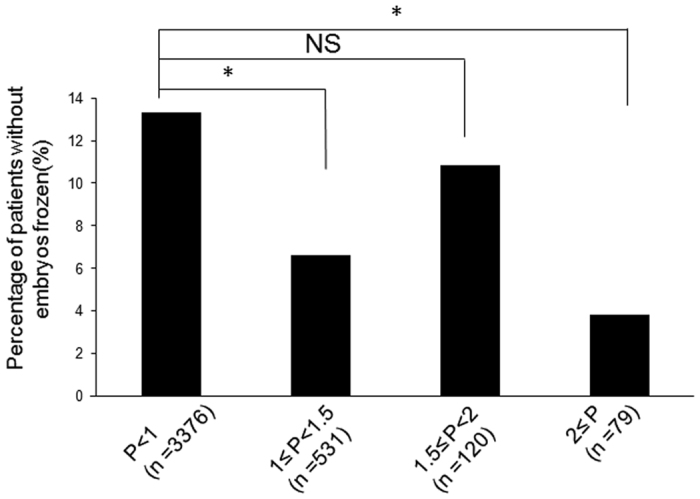
The Percentage of patients without embryos frozen in the groups with P < 1 ng/mL, P between 1–1.5 ng/mL, P between 1.5–2 ng/mL and P ≥ 2 ng/mL on the day of trigger. Asterisks indicate P < 0.05.

No significant between-group differences existed in oocyte retrieval rates, fertilization rates, implantation rates, clinical pregnancy rates, and live birth rates based on FET (Tables 2 and 3). Interestingly, patients with P ≥ 2 ng/mL exhibited more high-quality embryos than patients with P < 1 ng/mL (3.08 VS 2.33, P = 0.027) (Table 2). Moreover, the percentages of patients without embryos frozen were significantly lower in the groups with P between 1–1.5 ng/mL and P ≥ 2 ng/mL than that in the groups with P < 1 ng/mL (Fig. 3). To analyze the association between the COS variables involved in increased P levels, we performed a multivariate logistic regression analysis using P = 1 ng/mL as the threshold. The total hMG dose (P = 0.001), number of oocytes (P < 0.001), E2 value on the day of trigger (P < 0.001), FSH level on the day of trigger (P < 0.001), and LH level on the day of trigger (P < 0.001) were all associated with increased P levels (Table 4).

We hypothesized that P elevation during hMG + MPA cycles was the result of ovarian stimulation and was driven by the high follicle stimulating hormone dosage, estradiol levels, and the number of oocytes.

Patients with polycystic ovarian syndrome (PCOS) were frequently complicated by ovarian hyperstimulation to gonadotropin therapy (hMG or FSH)^17^. Consistent with our results, the percentages of PCOS patients in the groups with P between 1–1.5 ng/mL, P between 1.5–2 ng/mL and P ≥ 2 ng/mL were significantly higher than that in the group with P < 1 ng/mL (Fig. S1).

## Discussion

Subtle increases in serum P levels at the end of the follicular phase in COS cycles are a frequent phenomenon in GnRH analogue/antagonistic COS cycles. Evaluation of the effect of P elevation on the day of trigger is helpful when assessing the importance of its prevention^18^. In hMG + MPA IVF/ICSI treatment cycles, the incidence and effect of elevated P on the trigger day are still unknown. Here, we analyzed 4106 hMG + MPA IVF/ICSI treatment cycles to evaluate the incidence and effect of elevated P on the day of trigger. We found that 17.78% (730/4106) of the cycles showed elevated P (≥1 ng/mL), indicating that subtle P elevation was a frequent phenomenon in the hMG + MPA IVF/ICSI treatment cycles.

The elevation of P was probably a result of granulosa cells that started the process of luteinization induced by the premature LH increase. A premature LH increase was defined as LH ≥ 10 mIU/mL^19^. However, LH values gradually decreased during ovarian stimulation in the hMG + MPA treatment cycles, and the average LH level on the trigger day was significantly lower than the basal LH values in patients with P between 1–1.5 ng/mL and 1.5–2 ng/mL (1.78 VS 4.08, P < 0.05 and 2.10 VS 4.21, P < 0.05). A recent study showed that the increase in P in the late follicular phase was unrelated to any luteinizing process attributable to effects in the circulation or sensitization of follicular cells to LH^20^. Moreover, if the follicles of patients who exhibit P elevation start the process of luteinization, the resulting oocytes from these patients would be expected to be of lower quality^21^,^22^. However, the present study showed that the P elevation on the day of trigger did not have a negative effect on oocyte and embryo quality, which was consistent with the results of previous study. These data suggested that the elevated P levels observed in the hMG + MPA treatment cycles were not due to premature luteinization.

In the present study, we noted that FSH levels at the beginning of stimulation were higher in patients with P < 1.0 ng/mL than those in patients with P between 1–1.5 ng/mL, between 1.5–2 ng/mL, and ≥2 ng/mL; however, the mean serum FSH level on the day of trigger in the patients with P < 1 ng/mL was 15.03 mIU/mL, which was lower compared with the mean FSH levels of 16.59 mIU/mL, 16.15 mIU/mL, and 16.35 mIU/mL detected in the patients with P between 1–1.5 ng/mL, between 1.5–2 ng/mL, and ≥2 ng/mL, respectively. Moreover, the total dose of HMG administered in the patients with P between 1–1.5 ng/mL, between 1.5–2 ng/mL, and ≥2 ng/mL was higher than the dose used for patients with P < 1 ng/mL. These data indicated that the elevation of P was likely a consequence of activation of other pathways by FSH.

To evaluate the effect of P elevation on the day of trigger, we analyzed the association between P elevation and oocyte retrieval/fertilization rates. No significant differences in oocyte retrieval/fertilization rates were found between the groups with different P levels on the trigger day. These results indicated that P elevation did not have any negative effects on oocyte quality.

A recent study showed that elevated P at a threshold of 1.5 ng/mL was independently associated with a decreased chance of pregnancy in low to normal responders but not in high responders when using an rFSH/GnRH antagonist protocol based on fresh ET^15^. The negative effect of elevated P is believed to be exerted at the level of the endometrium rather than through affecting embryo quality. A retrospective analysis of more than 10,000 agonist cycles showed that the pregnancy rate in FET cycles was significantly higher than that in fresh ET cycles at higher P levels^23^. Here, we found that the group with P < 1 ng/mL had highest percentage of patients without embryos frozen. There were no significant difference in the implantation rate, clinical pregnancy rates and live birth rates based on FET between groups. These results indicated that the elevated P on the trigger day had no negative effective on the embryo quality. Interestingly, we noted that the implantation rate, pregnancy rate and live birth rate were higher in the groups with elevated P than those in the group with P < 1 ng/mL, though these differences did not reach the level of significance. Moreover, most of the patients with higher P on the trigger day were strong ovarian responders to COS and generated more high quality embryos. We hypothesized that performing FET cycles might lead to better pregnancy outcomes for patients with higher P levels.

## Methods

### Study population and design

This is a retrospective analysis of a cohort of IVF/ICSI cycles (n = 4106) in 4106 women performed at the Department of Assisted Reproduction of the Ninth People’s Hospital of Shanghai Jiaotong University School of Medicine during the period from November 2013 to June 2015. The criteria for inclusion were females with ages 20–38 years with a body mass index (BMI) of 18–30 kg/m^2^ and a basal FSH <10 mIU/mL. Only the first IVF/ICSI cycle treated with hMG + MPA of each woman was included. Patients underwent COS using hMG (150–225 IU/d; Anhui Fengyuan Pharmaceutical Co, Anhui, China) and MPA (4 mg, 8 mg, or 10 mg/d) from menstrual cycle (MC) day 3 as previously described^14^. The final stage of oocyte maturation was triggered when there were more than 3 dominant follicles reached 18 mm in diameter, using triptorelin (0.1–0.2 mg; decapeptyl, Ferring Pharmaceuticals) or cotriggered via SC (subcutaneous) injections of triptorelin (0.1–0.2 mg) and hCG (1,000–5000 IU; Lizhu Pharmaceutical Trading Co.). Transvaginal ultrasound–guided oocyte retrieval was conducted 34–36 hours after trigger administration. All follicles with diameters greater than 10 mm were retrieved. Fertilization of the aspirated oocyte was performed *in vitro* by either conventional insemination or ICSI depending on the semen parameters. Embryos were examined for the number and regularity of blastomeres and the degree of embryonic fragmentation on the third day according to the criteria of Cummins *et al.*^24^. All good-quality embryos (including grade 1 and grade 2 8-cell embryos) were frozen by vitrification on the third day after oocyte retrieval. Only non-top quality embryos were placed in extended culture until they reached the blastocyst stage. During this stage, only blastocysts with good morphology were frozen on day 5 or 6. The vitrification procedure for freezing cleavage-stage embryos and blastocysts was previously described^14^. For thawing, solutions of 1, 0.5, and 0 M sucrose were used sequentially as cryoprotectant dilutions. All vitrification and warming steps were performed at room temperature except for the first warming step, which was conducted at 37 °C.

Embryo and endometrium synchronization with FET were performed using the previously described method^14^,^25^. Briefly, natural FET cycles were used for women with regular menstrual cycles and letrozole was administered to patients with irregular menstrual cycles. In total, 3837 FET cycles were performed in this study. Among these FET cycles, 1.04% (40/3837) FET cycles were lost to follow-up because the patients changed their phone number and could not be contacted (Table 3).

### Hormone Analysis

Serum FSH, LH, E2, and P were measured on MC3, the trigger day, and the day after trigger (approximately 10 hours after the injection of GnRHa and/or hCG). Hormone levels were measured using chemiluminescence (Abbott Biologicals B.V.). The lower limits of sensitivity were as follows: FSH, 0.06 IU/L; LH, 0.09 IU/L; E2, 10 pg/mL; and P, 0.1 ng/mL. The upper limit of the E2 measurement was 5,000 pg/mL. The E2 values were recorded as 5,000 pg/mL if the E2 level on the trigger day or the day after trigger was higher than the upper limit.

### Statistical Analysis

The live birth rate was defined as the proportion of patients exhibiting live birth among all transfer cycles. The primary outcome measure was the live birth rate. Clinical pregnancy was defined as the presence of a gestational sac with fetal heart activity during ultrasound examination 7 weeks after FET. The implantation rate was defined as the number of gestational sacs divided by the number of transferred embryos. A logistic regression model was performed to quantify the effect of related factors on the level of P ≥ 1. The following possible factors were considered: female age, BMI, number of oocytes, total hMG dose, total hMG dose, duration of stimulation (d), MPA dose, FSH level on the day of trigger, and LH level on the day of trigger. All of these factors were introduced into the regression model. The data in the table are presented as the mean and 95% confidence interval (CI) of the mean. The chi-square test was used for categorical variables. Comparisons between groups were analyzed by ANOVA followed by an appropriate post hoc test. P < 0.05 was considered statistically significant. All data were analyzed using the Statistical Package for the Social Sciences for Windows (ver. 22, SPSS Inc.).

## Additional Information

**How to cite this article**: Lu, X. *et al.* Elevated progesterone on the trigger day does not impair the outcome of Human Menotrophins Gonadotrophin and Medroxyprogesterone acetate treatment cycles. *Sci. Rep.* **6**, 31112; doi: 10.1038/srep31112 (2016).

## Supplementary Material

Supplementary Information

## Figures and Tables

**Table 1:** Baseline patient characteristics.

Characteristics	P (ng/mL)				P Value
	<1 (n = 3376)	1–1.5 (n = 531)	1.5–2 (n = 120)	≥2 (n = 79)	
Age of women, mean (95%CI)	31.48(31.36–31.60)	30.56(30.26–30.85)	30.67(30.10–31.24)	30.18(29.38–30.98)	<0.05^a^
BMI of women, mean (95%CI)	21.88(21.79–21.96)	21.51(21.30–21.71)	21.41(20.99–21.84)	21.52(20.91–22.45)	<0.05^b^
Basic FSH (mIU/mL), mean (95%CI)	5.79(5.75–5.84)	5.22(5.12–5.31)	4.89(4.65–5.14)	4.77(4.49–5.05)	<0.001^a^
Basic LH (mIU/mL), mean (95%CI)	3.45(3.39–3.51)	4.08(3.88–4.28)	4.21(3.79–4.63)	4.54(3.92–5.16)	<0.05^a^
Basic E2 (pg/mL), mean (95%CI)	38.55(35.13–41.98)	34.63(33.00–36.25)	37.78(33.85–41.70)	37.96(33.77–42.15)	NS
Basic P (ng/mL), mean (95%CI)	0.31(0.30–0.32)	0.36(0.28–0.44)	0.47(0.23–0.76)	0.39(0.26–0.52)	NS

Data are presented as the means and 95% confidence interval. BMI: body mass index.

^a^Indicates group with P < 1 ng/mL versus groups with P between 1–1.5 ng/mL, between 1.5–2 ng/mL and ≥2 ng/mL.

^b^Indicates group with P < 1 ng/mL versus groups with P between 1–1.5 ng/mL and between 1.5–2 ng/mL.

**Table 2:** Ovarian stimulation characteristics and outcomes.

Characteristic	P (ng/mL)				P Value
	<1 (n = 3376)	1–1.5 (n = 531)	1.5–2 (n = 120)	≥2 (n = 79)	
Total hMG dose (IU),mean (95%CI)	1931.95(1917.05–1946.85)	2069.52(2032.12–2106.92)	2100.00(2041.20–2158.80)	2081.49(2011.84–2151.14)	<0.001^a^
Duration of stimulation (d), mean (95%CI)	8.92(8.86–8.92)	9.35(9.22–9.48)	9.51(9.28–9.74)	9.57(9.31–9.84)	<0.001^a^
Number of oocyte aspirated. mean (95%CI)	15.14(14.82–15.46)	25.62(24.58–26.66)	30.61(28.26–32.96)	30.74(27.57–33.91)	<0.001^a^
Number of oocyte retrieved, mean (95%CI)	10.38(10.15–10.60)	17.99(17.25–18.73)	20.39(18.85–21.94)	19.84(17.59–22.10)	<0.001^a^
Oocyte retrieval rate, mean (95%CI)	70.45(69.74–71.16)	72.01(70.45–73.57)	68.79(65.42–72.17)	66.89(62.47–70.93)	NS
Normal fertilization rate, mean (95%CI)	89.79(89.18–90.39)	90.00(88.88–91.13)	88.93(86.37–91.49)	89.38(85.43–93.33)	NS
Embryos frozen, mean (95%CI)	4.15(4.06–4.24)	5.97(5.69–6.26)	6.61(5.90–7.32)	6.43(5.55–7.31)	<0.001^a^
Top-quality embryos, mean (95%CI)	2.33(2.21–2.45)	2.87(2.59–3.22)	2.44(1.96–2.93)	3.08(2.03–4.13)	<0.05^b^
Hormones on day of trigger					
FSH (mIU/mL), mean (95%CI)	15.03(14.89–15.17)	16.59(16.24–16.95)	16.15(15.48–16.81)	16.35(16.12–17.29)	<0.001^a^
LH (mIU/mL), mean (95%CI)	1.74(1.69–1.80)	1.78(1.66–1.90)	2.10(1.65–2.55)	3.98(1.29–6.67)	<0.001^b^
E2 (pg/mL), mean (95%CI)	2844.63(2794.82–2894.44)	4492.73(4387.56–4597.90)	4838.66(4728.63–4948.68)	4412.58(4141.50–4683.67)	<0.001^a^

Data are presented as the means and 95% confidence interval.

^a^Indicates group with P < 1 ng/mL versus groups with P between 1–1.5 ng/mL, P between 1.5–2 ng/mL and P ≥ 2 ng/mL.

^b^Indicates group with P < 1 ng/mL versus group with P ≥ 2 ng/mL.

**Table 3:** Outcomes of FET cycles.

Variable	P (ng/mL)				P Value
	<1	1–1.5	1.5–2	≥2	
Implantation rate (%)	33.69(1894/5622)	34.57(355/1027)	36.48(85/233)	37.50%(57/152)	>0.05
Clinical pregnancy rate (%)	46.70(1444/3092)	49.07(265/540)	50(62/124)	49.38(40/81)	>0.05
Live birth rate (%)	41.92(1283/3060)	45.69(244/534)	47.15(58/123)	48.75(39/80)	>0.05
Lost to follow-up(%)	1.04(32/3072)	1.11(6/540)	0.81(1/124)	1.23(1/81)	>0.05

**Table 4:** Multivariate logistic regression of factors related to elevated P.

Factor	*OR* [95% CI]	P value
Age–yr	1.021 [0.993,1.050]	0.140
BMI-kg/m^2^	1.009 [0.965, 1.054]	0.703
Number of oocytes	1.051(1.038–1.063)	<0.001
Total hMG dose	1.001(1.000–1.001)	0.001
Duration of stimulation (d)	0.987(0.850–1.145)	0.858
MPA dose	0.998(0.995–1.002)	0.324
FSH level of day of trigger	1.106(1.075–1.139)	<0.001
LH level of day of trigger	1.123(1.060–1.191)	<0.001
E2 on day of trigger	1.001(1.001–1.001)	<0.001

OR, odds ratio; CI, confidence interval.
