# Supplementary material for: Elevated progesterone on the trigger day does not impair the outcome of Human Menotrophins Gonadotrophin and Medroxyprogesterone acetate treatment cycles
Source: Sci Rep. 2016 Aug 8;6:31112. doi: 10.1038/srep31112 (PMC4976389; doi:10.1038/srep31112)
Supplement: Supplementary Information [file srep31112-s1.pdf]

Supplementary data

Elevated progesterone on the trigger day does not impair the outcome of Human Menotrophins Gonadotrophin and Medroxyprogesterone acetate treatment cycles

Xuefeng Lu1, Qiuju Chen[1], Yonglun Fu[1], Ai Ai[1], Qifeng Lyu[1], Yan Ping Kuang[1, *]

[1] Department of Assisted Reproduction, Shanghai Ninth People's Hospital, Shanghai Jiaotong University School of Medicine, 639 Zhizaoju Rd, Shanghai 200001, China

Abbreviated title: Elevated progesterone on the day of trigger in hMG+MPA treatment cycles.

Key Terms： Elevated progesterone, Infertility, Controlled ovarian stimulation， Medroxyprogesterone acetate

[*]**Corresponding author:** Yanping Kuang, M.D and Ph.D

Department of Assisted Reproduction, Shanghai Ninth People's Hospital, Shanghai Jiaotong University School of Medicine, 639 Zhizaoju Rd, Shanghai 200001, China.

Tel: +86-21-23271699-5539

Fax: +86-21-53078108

E-mail: kuangyanp@126.com

Trial registration number: NA.

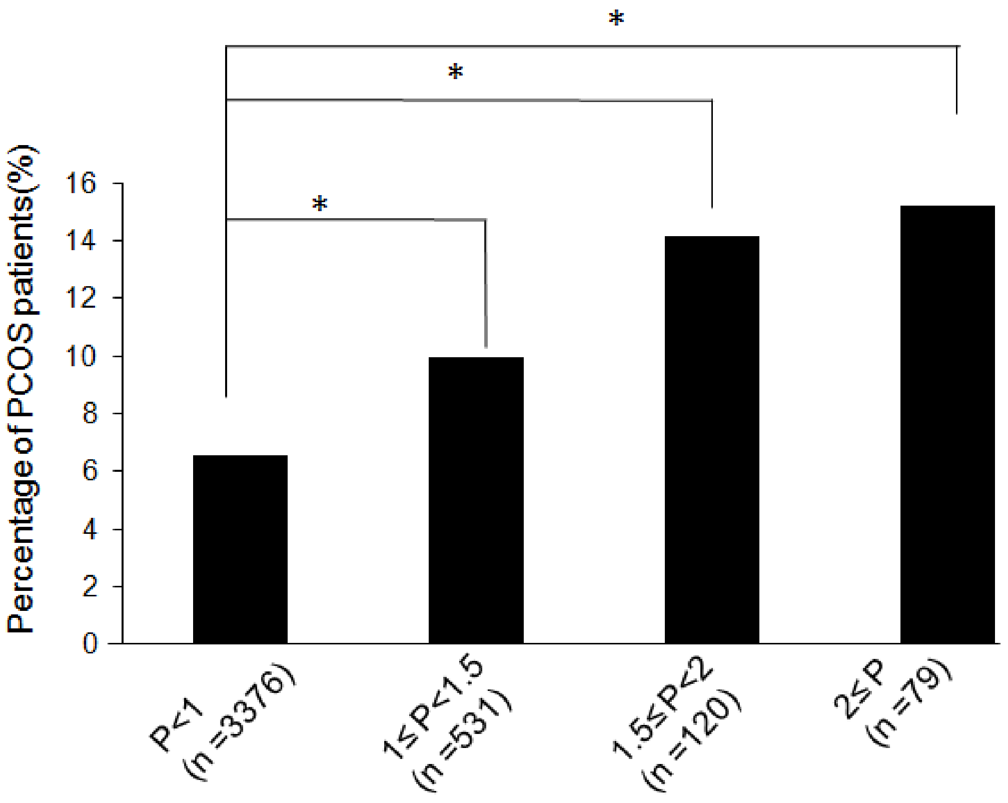

Figure. S1. The percentage of PCOS patients in the groups with P<1 ng/mL, between 1-1.5 ng/mL, between 1.5-2 ng/mL and ≥2 ng/mL on the day of trigger. The asterisks indicate P<0.05.
